# The Extracellular Matrix and Pancreatic Cancer: A Complex Relationship

**DOI:** 10.3390/cancers10090316

**Published:** 2018-09-06

**Authors:** Maximilian Weniger, Kim C. Honselmann, Andrew S. Liss

**Affiliations:** Department of Surgery, Massachusetts General Hospital and Harvard Medical School, Boston, MA 02114, USA; mweniger@mgh.harvard.edu (M.W.); khonselmann@mgh.harvard.edu (K.C.H.)

**Keywords:** collagen, hyaluronan, tissue stiffness, matrisome

## Abstract

Pancreatic ductal adenocarcinoma (PDAC) has an extraordinarily dense fibrotic stroma that impedes tumor perfusion and delivery of anticancer drugs. Since the extracellular matrix (ECM) comprises the bulk of the stroma, it is primarily responsible for the increased interstitial tissue pressure and stiff mechanical properties of the stroma. Besides its mechanical influence, the ECM provides important biochemical and physical cues that promote survival, proliferation, and metastasis. By serving as a nutritional source, the ECM also enables PDAC cells to survive under the nutrient-poor conditions. While therapeutic strategies using stroma-depleting drugs have yielded disappointing results, an increasing body of research indicates the ECM may offer a variety of potential therapeutic targets. As preclinical studies of ECM-targeted drugs have shown promising effects, a number of clinical trials are currently investigating agents with the potential to advance the future treatment of PDAC. Thus, the present review seeks to give an overview of the complex relationship between the ECM and PDAC.

## 1. Introduction

By 2030, pancreatic ductal adenocarcinoma (PDAC) will be the second leading cause of cancer-associated deaths in the United States, yet patients continue to face a dismal prognosis owing to early local and distant spread of tumor [1,2,3]. PDAC is characterized by a pronounced resistance to radiation, cytotoxic, and molecularly targeted therapies, such that only small subsets of patients benefit from present-day systemic treatments [4,5]. Here, recent clinical trials using FOLFIRINOX in patients with good performance status have demonstrated unprecedented increases of survival in both metastatic and potentially curable PDAC [4,6]. Patients with favorable comorbidity profiles also benefit from the addition of nab-paclitaxel to gemcitabine [7]. However, only roughly one quarter to one-third of patients with metastatic disease responds to these therapies [4,7], and none of the currently available treatment options provides long-term survival for the average patient [7]. 

The chemo- and radiotherapeutic resistance of PDAC is thought to be mediated, in large part, by its prominent stroma, composed of a variety of non-neoplastic cell types and extracellular matrix (ECM). The deposition of abundant amounts of ECM is termed desmoplastic reaction and exerts mechanical as well as biochemical effects on PDAC cells [8]. In addition to directly affecting the biology of PDAC cells, both the mere amount of ECM and water retention by ECM glycoproteins result in high interstitial fluid pressure, thereby impairing tumor perfusion and thus delivery of antitumor drugs [9,10]. This effect is further aggravated by reduced tumor vessel density, making cytotoxic therapy of PDAC extraordinarily challenging [9].

With respect to its cellular components, the PDAC stroma is dominated by cancer-associated fibroblasts (CAFs.) While a heterogeneous population, CAFs are largely composed of activated pancreatic stellate cells (PSC) [11]. In normal pancreatic tissue, quiescent PSCs reside at the basolateral aspect of pancreatic acinar cells and synthesize ECM proteins and ECM-degrading enzymes [12,13]. Therefore, PSCs are thought to regulate ECM turnover by maintaining a balance between ECM synthesis and degradation [13]. Once PSCs become activated, however, the equilibrium shifts, causing the accumulation of large amounts of ECM proteins [11,13].

This transition from quiescent to activated PSC is accompanied by significant morphological changes in the cytosol and cell shape. While in their quiescent state PSCs exhibit abundant lipid droplets containing vitamin A, these droplets disappear upon activation and the cells form contractile stress fibers, resulting in a spindle-shaped, myofibroblast-like phenotype [13,14]. Strikingly, activation of the vitamin A receptor reduces PSC contractility and counteracts PSC activation [15]. Similarly, vitamin D agonists revert PSC activation, but it is unclear whether vitamin D is present in PSC lipid droplets [16].

PDAC cells are an important driver of ECM production by PSC. PDAC cells secrete sonic hedgehog (SHH), which functions as a signaling molecule to attract and activate PSCs [17,18]. More precisely, PSCs show enhanced migration towards SHH overexpressing PDAC cells and treatment with SHH-inhibiting antibodies significantly reduces tumor desmoplasia in mice orthotopically implanted with PDAC cells [18]. Independent of SHH, PDAC cells secrete fibroblast growth factor 2, platelet-derived growth factor, and transforming growth factor beta 1 (TGFβ1), which promote PSC activation and collagen synthesis [19,20]. Interestingly, PSCs not only express receptors for TGFβ1 and respond to PDAC derived TGFβ1 signaling, they also express TGFβ1 themselves [21]. Thus, once activated, PSCs become part of a self-sustaining pathological cycle that perpetuates PDAC fibrosis. 

Data demonstrating a key role for CAFs in mediating ECM production have triggered a multitude of studies attempting to deplete CAFs in PDAC tumors. However, as highlighted by a number of preclinical studies [22,23,24] and the halted trial on saridegib [13], therapies that target and deplete stromal cells result in a more aggressive disease. Hence, efforts to completely deplete the stroma remain controversial. In this respect, approaches that target and modify the ECM are being intensively studied in both preclinical and clinical research. The complex network of ECM proteins is fundamental to tissue homeostasis in health [25] and crucially influences tumorigenic cell features including growth, differentiation, and metastasis in cancer [26]. By serving as a reservoir for signaling molecules [27] and influencing cell signaling by transducing mechanical forces [25] and direct binding to cell surface receptors [28], the ECM plays a key role in PDAC. This review aims to summarize current evidence for the role of the ECM in PDAC and to highlight potentially targetable pathways. Furthermore, by analyzing ongoing clinical trials of ECM-targeted therapy, future clinical therapeutic options are discussed. 

## 2. Composition and Role of the ECM

The ECM is a three-dimensional non-cellular network that is present in every organ and vital for life. Historically, efforts to define the composition of the ECM reach back as far as the 1950s [29], and recent genetic and bioinformatic studies [30], as well as mass spectrometry analysis [31], have led to an improved understanding of this meshwork of large cross-linked proteins [31,32]. The ECM may be organized either as an interstitial matrix or as specialized forms, such as the basement membrane or vascular endothelium [33]. In addition to ensuring tissue integrity, the ECM is crucially involved in cell signaling by supplying components that bind directly to cell surface receptors and ECM-derived peptides (matrikines) that have been liberated after proteolytic cleavage of bioactive fragments of ECM proteins [34]. In doing so, the ECM may dictate the fate of cells and organs. Besides biochemical signaling, the mechanical properties of the ECM provide important physical cues to cells that, in turn, influence intracellular signaling cascades [8]. The importance of the ECM is highlighted by both cancerous and connective tissue disorders. Mutations in ECM genes are associated with conditions like Marfan and Ehlers–Danlos syndromes [35,36]. Ehlers–Danlos syndrome comprises a broad range of mutations in genes encoding fibrillar collagens or enzymes that regulate their synthesis. In the classical presentation of this disease, mutations in procollagen V (COL5A1/COL5A2) prevent the assembly of collagen I and V heterotrimers, resulting in joint hypermobility, skin hyperextensibility, and widened atrophic scars [35]. Marfan syndrome is caused by mutations in the glycoprotein fibrillin-1 (FBN1) [36]. These mutations result in insufficient fibrillin-1 synthesis and loss of ECM architecture, leading to clinical defects dominated by thoracic aortic aneurysms and dissections [36]. Furthermore, ECM remodeling has also been identified as a consequence of or increased risk for malignant transformation of hepatic [37], pulmonary [38], and pancreatic cells [11]. More precisely, hepatocellular carcinoma is the most common cause of death in patients with liver cirrhosis [37], the incidence of lung cancer is increased fourfold in patients with idiopathic pulmonary fibrosis in comparison to patients with pulmonary emphysema [38], and as emphasized before, the abundant amount of ECM is a hallmark of PDAC [11].

Collagens are by far the most abundant and well-characterized component of the ECM in PDAC tumors. Currently, 28 different types of collagen have been described [39], representing the main thrust of research on ECM proteins. Therefore, this review focuses largely on collagens and their interactions with PDAC cells, highlighting the various mechanisms by which ECM can influence tumor biology. Amongst others, the basement membrane collagens include collagen IV, XV, and laminin [40], while collagen I, III, and V are located in the interstitial space [41]. In PDAC, collagen I is responsible for the majority of the desmoplastic reaction [42,43,44,45]. Importantly, the desmoplastic reaction leads not only to quantitative changes in the total amount of ECM but also to qualitative changes. By disrupting the normal architecture of the basement membrane, PDAC cells are exposed to increasing amounts of interstitial collagens, which may have protumorigenic effects [42,44]. In this respect, the deposition of high levels of collagen I has been associated with reduced survival [46].

While multiple lines of evidence show that interstitial collagens, such as collagen I, foster protumorigenic features, including invasion and EMT, the basement membrane type collagen XV hinders these cell features [47]. As a component of the ECM that has antitumor effects, expression of collagen XV is reduced in basement membranes of aggressive colon carcinomas [48] and is lost during the progression from carcinoma in-situ to invasive carcinoma in breast cancer [49]. Thus, unlike collagen I, expression of collagen XV is decreased during tumor progression. Additionally, overexpression of collagen XV reduces the migratory capabilities of PDAC cells in matrices rich in collagen I [47]. Furthermore, two major components of the basement membrane have been demonstrated to have a substantial, yet different impact on survival. Here, laminin content <25% in basement membranes is associated with decreased long-term survival [50]. In contrast to the beneficial impact of laminin, patients with high postoperative levels of circulating collagen IV exhibit dramatically reduced survival after curative resection of PDAC [51]. Collagen IV shows high expression levels in PDAC stroma and promotes proliferation and migration of PDAC cells [52]. Further, PDAC cells produce collagen IV, which protects PDAC cells themselves from serum deprivation-induced apoptosis [52]. Thus, collagens have important effects on PDAC cells; however, even within the same group of collagens, these effects may differ. 

## 3. Cell Signaling via Collagens

One of the mechanisms by which collagens contribute to PDAC biology is by functioning as signaling molecules, or ligands, for integrin receptors on the surface of PDAC cells [52]. Integrins are transmembrane glycoproteins that are composed of heterodimers of α- and β-subunits, and the combination of these subunits dictates specificity to various collagens. In this regard, integrin α2β1 has a high affinity for both collagen I and V and also binds weakly to collagen IV [53]. Conversely, integrin α1β1 binds weakly to collagen I but has a high affinity for collagen IV [53,54]. Binding of collagen I to integrin has been shown to promote the proliferation and migration of PDAC cells and to prevent apoptosis [52,55]. Similar effects are exerted by collagen V, which has been shown to foster adhesion, proliferation, migration, and viability in several PDAC cell lines after binding to α2β1 integrin receptors [56,57]. Notably, knockdown of integrin-β1 not only inhibits adhesion of PDAC cells to collagen but also reduces tumor proliferation and abrogates metastasis in an orthotopic mouse model of human PDAC cells [58]. 

When collagen is bound to the integrin receptor, important downstream signaling events are activated. For example, the migration of PANC-1 and UlaPaCa cells along collagen I gradients is mediated by the activation of the focal adhesion kinase (FAK) pathway by collagen I-integrin signaling [59]. In PDAC, collagen I-mediated activation of the FAK pathway also increases colony formation, clonogenic growth, and self-renewal [60,61]. Additionally, activation of FAK by collagen I may regulate epithelial to mesenchymal transition (EMT). Collagen I-mediated activation of FAK induces β-catenin phosphorylation, thereby leading to disruption of the E-cadherin complex and activation of the canonical WNT signaling pathway [62,63]. Loss of the E-cadherin complex and subsequent loss of cell-cell adhesions is an important step in metastasis and EMT [64]. Activation of the FAK-pathway not only results in loss of E-cadherin but also in increased expression of N-cadherin, ultimately leading to increased neural invasion and migration of PDAC cells [59,65,66]. Thus, EMT may be driven by the composition of the ECM, whereby microenvironments rich in collagen I promote a more aggressive phenotype.

In addition to integrin-mediated signaling, collagens bind to discoidin receptor 1 (DDR1), a dimeric transmembrane tyrosine kinase [67] which is overexpressed in PDAC [68,69], as well as breast cancers [70]. Binding of collagen I to DDR1 activates FAK-related protein tyrosine kinase (PYK2), resulting in the expression of N-cadherin [66]. While DDR1 is thought to be an important receptor for collagens, PDAC cells also utilize this receptor by expressing transmembrane-4-L-six-family member 1 (TM4SF1), which after binding to DDR1, results in the formation of invadopodia and induces cell migration [71]. Furthermore, binding of TM4SF1 to DDR1 induces the expression of matrix metalloproteinase (MMP) 2 and 9, enzymes involved in the degradation of the ECM [71,72]. Consistent with this, TM4SF1 has been demonstrated to promote migration and invasion in liver [73], breast [74], colorectal [75], and pancreatic cancer cell lines [72]. Moreover, knockdown of TM4SF1 results in reduced liver and pulmonary metastases in an MIA PaCa-2-derived orthotopic xenograft mouse model of PDAC [72]. Interestingly, the effects of collagen I and TM4SF1 may not be entirely independent. Besides binding to DDR1 itself, TM4SF1 has been shown to support clustering of collagen I-bound DDR1 receptors in breast cancer [76]. 

Extracellular signaling ultimately results in altered gene expression. Not surprisingly, collagens exert profound epigenetic effects on PDAC cells. When grown in collagen gels, PDAC cells show increased expression of the histone acetyltransferases p300, P300/CBP-associated factor, and GCN5 [77]. Accordingly, increased acetylation of the histones H3K9 and H3K27 is observed, all of which is associated with increased levels of gene expression. Additionally, high mobility group A2, an epigenetic regulator of proliferation, apoptosis, and DNA repair, shows increased levels of expression when PDAC cells are grown in collagen gels [78]. The signaling mechanisms that mediate these epigenetic alterations remain unclear and additional work is needed to determine the relative impact of collagen and 3D growth on these epigenetic changes. Table 1 summarizes the different effects of collagens on PDAC cells.

## 4. Structural Regulation of the ECM

By providing mechanical cues, such as tissue stiffness, the ECM can alter the properties of PDAC cells. In this regard, a stiff tumor stroma reduces tissue polarity, disrupts adherens junctions, and results in enhanced tumor cell proliferation [79]. Furthermore, the stiffness of the ECM has an important influence on EMT; stiff collagen matrices alter the expression of vimentin and E-cadherin and increase nuclear translocation of β-catenin in PDAC cells [80]. Cross-linked and thus stiffer collagen fibers have also been shown to be associated with enhanced MMP activity, which may be interpreted as a surrogate for increased invasive tumor properties [81]. Conversely, chemical inhibition of PDAC cell contractility results in decreased MMP activity, indicating that PDAC cells themselves can also influence the mechanical properties of ECM [81]. Consistent with these biological and biochemical changes, increased collagen fiber thickness and, thus, stiff tumor stroma are associated with poor patient survival [8].

Stiffening of the PDAC tumor stroma is achieved by the cross-linking of collagen fibers by lysyl oxidase (LOX), an extracellular amine oxidase that predominantly cross-links collagen I [82,83]. Expression of LOX is increased under hypoxic conditions and is a critical factor for metastasis [84]. Studies employing a genetically engineered mouse model of PDAC (Pdx1-Cre;Kras^G12D^;Trp53^R172H^) have demonstrated that systemic administration of a neutralizing antibody specific to LOX results in reduced proliferation of metastases and improved survival [82]. It is possible that some of these effects are due to activities of LOX beyond collagen, as inhibition of LOX not only results in reduced collagen cross-linking but also stromal collapse and improved vessel density [82]. 

Another enzyme that cross-links collagen I fibers in PDAC is tissue transglutaminase 2 (TG2) [85]. TG2 is induced by TGFβ and works by transferring acyl groups between glutamine and lysine residues [86]. While TG2 is expressed weakly in normal pancreatic tissue, TG2 expression levels are dramatically increased in PDAC [85]. PDAC cells express TG2 and secrete it into the ECM, where it not only cross-links collagen fibers but also stimulates cancer-associated fibroblasts to produce collagen I [85]. Additionally, TG2 secretion by PDAC cells and the resulting increases in ECM stiffness have boomerang effects on PDAC cells [85]. More precisely, cross-linked collagen activates Yes-associated protein (YAP) and transcriptional coactivator with a PDZ-binding motif (TAZ), (transcription factors found in PDAC cells), ultimately resulting in enhancement of proliferation [85] and EMT [80]. YAP/TAZ signaling is a key element in the response of cells to mechanical cues of their surrounding environment, which is highlighted by the increased nuclear localization of YAP/TAZ in response to increased ECM stiffness [87]. Notably, a recent bioinformatic analysis of mammary epithelial cells revealed that only gene expression signatures connected to YAP/TAZ were found to be associated with ECM stiffness [88]. Thus, YAP/TAZ may be a central hub in the transduction of mechanical ECM properties. 

While, in theory, deposition of enormous amounts of collagen around PDAC cells might hinder invasive growth and metastasis, 80% of patients present with locally advanced or metastatic disease. Not surprisingly, PDAC cells have mechanisms that help them overcome this fibrotic barrier. As such, MMPs are the main enzymes responsible for ECM degradation and remodeling and may pave the way for metastases [89]. There are 23 MMPs, each with specific targets within the ECM, but collectively MMPs degrade all structural components of the ECM [89]. Interestingly, collagen I has been shown to induce membrane type 1-MMP (MT1–MMP) expression, which suggests collagens can regulate the expression of enzymes involved in their remodeling [90]. MMPs can be inhibited by tissue inhibitors of matrix metalloproteinases (TIMPs), and the direction of ECM remodeling is dictated by the balance between MMPs and TIMPs [12,91]. TIMPs also have effects independent of MMP regulation. Irrespective of stromal density, increased expression of TIMPs promotes resistance to chemo- and radiotherapy and fosters proliferation of human and murine PDAC cell lines [92]. 

It is noteworthy that proliferation of Panc-1 cells is reduced significantly when MMP inhibitors are applied in collagen I-rich gels [93]. This finding is strengthened by the fact that these cells do not show changes of the cytoskeleton or cell shape in the absence of MMP activity [93]. ECM remodeling is also regulated by Rho-associated protein kinase (ROCK) signaling. ROCK proteins are kinases that regulate tissue contractility by controlling ECM remodeling and the contractility of actomyosin fibers [94]. In this regard, ROCK1 and ROCK2 promote the expression of MMP10 and 13, resulting in enhanced collagen degradation and local invasion [95]. Inhibition of ROCK not only resulted in reduced ECM degradation but also improved survival in a Pdx1-Cre;Kras^G12D^;Trp53^R172H^ mouse model of PDAC [95], suggesting that the ECM provides at least a partial protective effect in PDAC. 

## 5. ECM as a Nutritional Source

The proliferation of tumor cells requires a constant supply of nutrients. However, as the dense desmoplastic reaction restrains tissue perfusion, the influx of oxygen and nutrients is restricted [96]. As a mechanism to thrive under these precarious conditions, PDAC cells utilize macropinocytosis to acquire nutrients. KRAS-mutant PDAC cells (>90%) are characterized by membrane ruffling, which enables the PDAC cells to form macropinosomes, large vacuoles that may non-specifically take up extracellular molecules and transfer them to lysosomes for degradation [97,98]. The ability of PDAC to use this mechanism as a source of nutrients is highlighted by the continued growth of PDAC cell lines in the absence of essential amino acids when media is supplemented with albumin [96]. Consequently, inhibition of macropinocytosis inhibits the proliferation of PDAC cells [98]. Importantly, macropinocytosis may also allow PDAC cells to use the collagen-rich tumor microenvironment as a source of energy. A recent study revealed that PDAC cells metabolize collagen fragments under glucose-limited conditions [45]. This work showed that PDAC cells take up collagens using macropinocytosis when deprived of glucose and degrade them into amino acids, of which proline is then metabolized in the tricarboxylic acid cycle. 

## 6. Proteoglycans and Glycoproteins

Proteoglycans and glycoproteins are additional components of the ECM that have an important impact on tumor cells. Proteoglycans and glycoproteins are composed of core proteins that undergo post-translational glycosylation, which substantially shapes their conformation and cell signaling function [99]. In cancers, proteoglycans and glycoproteins are frequently subject to aberrant glycosylation resulting in structural and quantitative changes [100]. Commonly, cancer cells undergo alterations involving sialylation, branch-glycans, and core fucosylation [101]. Additionally, both *N*-glycosylation [102] and expression of the core proteins of periostin [102], fibulin 1 [102], and galectin 1 [103] have been found to be upregulated in PDAC [104,105]. Galectin 1 is expressed in several tumor types. It is involved in proliferation, invasion, angiogenesis, metastasis, and is linked to patient survival [105,106]. Furthermore, combining loss of galectin-1 with genetically engineered mouse models of PDAC (Ela-myc and Ela-Kras^G12V^p53^−/−^) resulted in diminished stromal activation and tumor cell proliferation, and increased infiltration of cytotoxic T-cells [107,108]. Thus, in accordance with the multitude of possible pre- and posttranslational modifications, glycoproteins have multifaceted roles in PDAC. This is further highlighted by fibronectin, which both shares similarities with collagens but also has its own distinct impact on PDAC biology. Similar to collagens, fibronectin binds to integrin receptors (α5β1), thereby activating the FAK pathway [109]. Furthermore, fibronectin has binding sites for collagens, making it a linker protein between collagens and integrins and supporting the role of collagens [110]. Besides these cooperative effects with collagens, fibronectin has been found to be a key factor in the resistance of PDAC to radiotherapy. Irradiation of PDAC cells induces their infiltration of the basement membrane, which is abrogated by application of either integrin α5β1 blocking antibodies or depletion of fibronectin [111]. In addition to its role in resistance to therapy, fibronectin supports the malignant biology of PDAC cells by stimulating proliferation [112] and production of reactive oxygen species [113]. Strikingly, fibronectin plays an important role in amplifying ECM synthesis by PSCs. By binding to the latent TGFβ binding protein, fibronectin allows for the release of active TGFβ, which in turn activates PSCs [19,114]. Accordingly, fibronectin is a key element of the ECM, both promoting malignant traits of PDAC cells and sustaining fibrogenesis. Similar to fibronectin, vitronectin is a major glycoprotein that binds to both integrins (α5β3) and collagens [110,115]. Involved in wound healing and hemostasis in health, vitronectin is overexpressed in PDAC and promotes cancer cell migration when combined with collagen I [116]. Promoting the malignant characteristics of PDAC cells further, vitronectin stimulates secretion of interleukin 8, promoting proliferation of PDAC cells [117,118]. Interestingly, vitronectin promotes expression of TGFβ in hepatic stellate cells [119], providing another example of how ECM proteins maintain their own synthesis and the overall desmoplastic reaction. 

Proteoglycans also play an important role in PDAC biology, but their contribution appears to be both pro- and anti-tumorigenic. While biglycan (proteoglycan-I) negatively correlates with patient prognosis in PDAC [120], patients with stromal expression of lumican, a small leucine-rich proteoglycan [121], have markedly improved survival and a reduced occurrence of metastasis [122]. Several of mechanisms may contribute to these improved biological outcomes. Exposure to extracellular lumican renders PDAC cells into a quiescent state by inducing G0/G1 cell cycle arrest [123]. Furthermore, extracellular lumican induces epidermal growth factor receptor internalization, thereby inhibiting AKT and mitogen activated protein kinase (MAPK) signaling [122,123]. 

**Figure 1:**
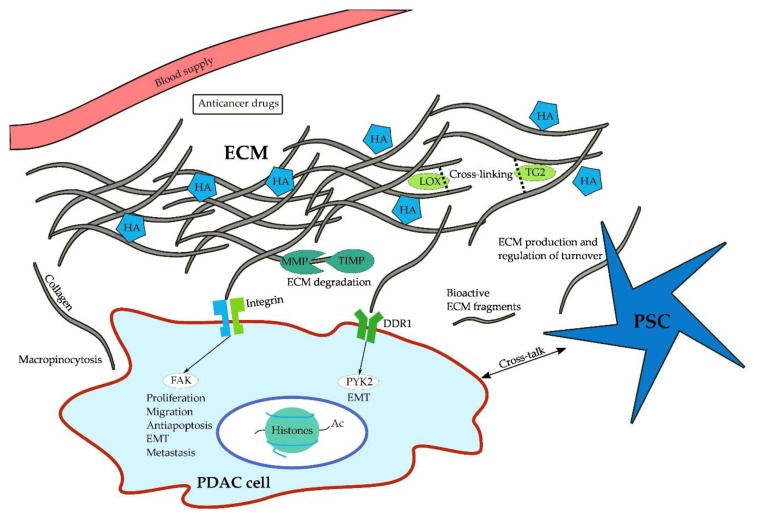
Interactions between extracellular matrix, cancer cells, and pancreatic stellate cells. Forming a dense meshwork of collagen fibers around pancreatic ductal adenocarcinoma (PDAC) cells, the extracellular matrix (ECM) impairs tumor perfusion and penetration by anticancer drugs. On top of these mechanical effects, collagen fibers bind to cell surface receptors, activating intracellular signaling pathways that induce protumorigenic programs. Here, both the biochemical and biomechanical effects of the ECM may be amplified by collagen cross-linking and hyaluronic acid (HA). In addition to interacting with the ECM itself, PDAC cells also communicate with pancreatic stellate cells (PSCs), which steer the turnover of the ECM. *DDR1, dimeric discoidin receptor 1; ECM, extracellular matrix; FAK, focal adhesion kinase; HA, hyaluronic acid; LOX, lysil oxidase; MMP, matrix metalloproteinase; PSC, pancreatic stellate cells; Pyk2, FAK-related protein tyrosine kinase; TG2, tissue transglutaminase 2; TIMP, tissue inhibitor of matrix metalloproteinases*.

Proteoglycans often bind non-covalently to hyaluronic acid (HA), a non-sulfated glycosaminoglycan that retains significant amounts of water and thereby contributes to the gel-like character of the interstitial fluid [124]. First occurring in pre-neoplastic pancreatic intraepithelial neoplasia (PanIN) lesions, HA is expressed in abundant amounts in the ECM [123] and following application of PEGPH20, an HA-degrading enzyme, intratumoral tissue pressure is decreased in PDAC mouse models (Pdx1-Cre;Kras^G12D^;Trp53^R172H^) [125,126]. Further studies using PEGPH20 have demonstrated that depletion or reduction of hyaluronic acid results in improved tumor perfusion and thus improved delivery of cytotoxic therapy in PDAC mouse models [10,126,127]. Moreover, its importance is highlighted by its ability to promote cell survival, proliferation, and invasion via binding to CD44 [128,129,130,131,132] and the receptor for hyaluronic acid-mediated motility (RHAMM) [133]. In addition to PEGPH20, HA may also be targeted using angiotensin inhibitors, which have been shown to reduce stromal HA and collagen production [134]. However, HA is likely to require the help of collagen to induce an increase in tissue pressure. As shown by Chauhan and colleagues, the amount of HA does not correlate with vessel compression in collagen-poor tumors, whereas a strong effect is evident in collagen-rich tumor microenvironments [134]. Figure 1 illustrates the multiple interactions of PDAC cells with their microenvironment.

## 7. Strategies to Overcome the ECM as a Barrier to Drug Delivery

### 7.1. Using the ECM to Target Chemotherapies to the Tumor

In addition to a multitude of genetic aberrations that make molecular targeted therapy challenging in PDAC, antitumor therapy has been profoundly crippled by the chemoresistance of PDAC to these targeted therapies. Here, the fibrotic ECM and high interstitial fluid pressure conjoin to reduce vascular patency thereby impeding the delivery of antitumor drugs. Despite this, efforts have been made to utilize the properties of the ECM to target drugs to PDAC tumors. Table 2 provides an overview of currently active clinical trials using ECM-targeted drugs. Secreted protein acidic and rich in cysteine (SPARC) is frequently overexpressed in PDAC [135] and has been associated with impaired patient survival [136,137]. Owing to its albumin-binding properties [138], it has been postulated that high levels of SPARC may allow for the enrichment of tumors with nanoparticle albumin-bound (nab)-paclitaxel, thereby enhancing peritumoral drug delivery [139]. Further testing in randomized clinical trials demonstrated a survival benefit in patients treated with gemcitabine and nab-paclitaxel compared to gemcitabine monotherapy (metastatic PDAC, median survival 8.5 months vs. 6.7 months, *p* < 0.001) [7]. However, it is not clear whether this is attributable to SPARC-mediated effects. In this regard, the Metastatic Pancreatic Adenocarcinoma Clinical Trial (MPACT) trial, which compared nab-paclitaxel plus gemcitabine to gemcitabine monotherapy, failed to demonstrate an association between SPARC expression and overall survival (SPARC high vs. SPARC low, median survival 8.0 vs. 7.6 months, *p* = 0.903) [140]. Additionally, a study using a genetically engineered mouse model of PDAC (p48Cre;Kras^LSL-G12D^;Trp53^flox/+^) that was bred with SPARC-positive, and SPARC-negative mice did not find an association between intratumoral accumulation of nab-paclitaxel and SPARC expression [141]. 

### 7.2. Inhibiting ECM Production

As shown by both experimental [22,23] and clinical [13] studies, stromal depletion results in more aggressive disease and impaired patient outcomes. In this respect, modulation of the activation state of PSC, as opposed to depletion of these cells, is hypothesized to be a viable strategy. One approach has been to target the vitamin D receptor in PSCs. Here the vitamin D receptor serves as a regulator of PSC activation, and vitamin D receptor agonists revert PSCs to a quiescent state, reducing tumor fibrosis and enhancing delivery of chemotherapeutics [16]. Further preclinical studies have demonstrated that vitamin D agonists may also reduce EMT and cancer cell stemness in PDAC [142,143]. Several clinical trials investigating the vitamin D analog paricalcitol in both resectable and metastatic PDAC are currently recruiting patients (NCT03520790, NCT03415854, NCT02930902, NCT03331562, NCT03300921, NCT03519308), but their clinical impact remains to be determined. Similarly, PSCs also express retinoic acid receptors, which interact with all-trans retinoic acid (ATRA), a metabolite of vitamin A [15]. By binding to retinoic acid receptor β, ATRA inhibits PSC activation, reduces ECM remodeling, and diminishes the ability of PSC to sense external mechanical cues from a stiff ECM [15]. ATRA is currently being utilized in a dose-finding phase I trial, where it is administered as an adjunct to gemcitabine and nab-paclitaxel (NCT03307148) in locally advanced and metastatic PDAC.

Besides utilizing vitamin A and D agonists, further alterations in the composition of ECM might be achieved through inhibition of angiotensin, a profibrotic cytokine [144]. Inhibiting the renin-angiotensin system has been associated with improved patient survival in retrospective studies of gemcitabine in advanced PDAC [145,146]. On a molecular level, losartan, an angiotensin receptor blocker, has been shown to reduce expression of TGFβ, HA synthases 1–3, and collagen I by cancer-associated fibroblasts [134,147]. Furthermore, enalapril, another inhibitor of the renin-angiotensin system, combined with aspirin, has been demonstrated to delay progression from pre-neoplastic PanIN lesions to PDAC in a Pdx1-Cre;Kras^G12D^;Trp53^R172H^ mouse model of PDAC [148]. Currently, losartan is under investigation in two clinical trials on PDAC, one where it is administered in addition to intraoperative gemcitabine (NCT01276613) to analyze its effect on the intratumoral accumulation of gemcitabine, and one where it is combined with FOLFIRINOX and proton beam radiation to investigate the potential effects on progression-free survival in locally advanced PDAC (NCT01821729). 

### 7.3. Preventing PDAC Cells from using the ECM as a Nutritional Source

Since the ECM serves as a source of glucose and amino acids for PDAC cells under the meager conditions of the desmoplastic reaction, targeting macropinocytosis might block the ECM as a nutritional supply for PDAC [45]. As an inhibitor of lysosomal degradation, a key step in macropinocytosis, hydroxychloroquine (HQ) is currently being investigated in two phase I/II trials in both resectable and locally advanced or metastatic PDAC (NCT01506973 and NCT03344172), and initial findings from other early phase trials have already been published. Here HQ reduced CA19-9 in resectable patients [149], but patients with metastatic disease did not show any significant therapeutic efficacy [150]. 

### 7.4. Relieving Intratumoral Pressure

As outlined in the previous section, HA constitutes a major part of the ECM and is thought to contribute to diminished tumor perfusion in PDAC. Prompted by studies in mouse models of PDAC showing that degradation of HA results in both improved interstitial fluid pressure and delivery of cytotoxic therapy [10,126,127], PEGPH20 was investigated in phase I and II studies on patients with metastatic PDAC [151,152]. Here, patients receiving PEGPH20 plus gemcitabine and nab-paclitaxel benefited from improved median progression-free survival (6.0 vs. 5.3 months, *p* = 0.049) [151]. Moreover, patients with high levels of HA expression showed an improved objective response and median survival (11.7 vs. 9.7 months, *p* = 0.04). Based on this data, PEGPH20 is being investigated in the phase III Halo 301 trial (NCT02715804) in combination with gemcitabine and nab-paclitaxel, where potential survival benefits of PEGPH20 in patients with metastatic PDAC are analyzed. Besides the Halo 301 trial, several clinical trials using PEGPH20 are ongoing, one of which is the phase I trial NCT03481920. In this trial, PEGPH20 is used in combination with avelumab, an immunological check-point inhibitor, to analyze drug safety and the overall response rate in patients with metastatic or locally advanced PDAC.

### 7.5. Potential Novel Therapeutic Avenues

The abundance of oncogenic effects of collagens makes therapies that directly address collagen, its receptors, or downstream pathways a potentially attractive antitumor strategy. As such, a receptor of collagens that could be targeted is DDR1. DDR1 was effectively inhibited by the orally available small molecule kinase inhibitor 7rh, resulting in decreased tumor burden and improved response to concomitant chemotherapy in xenograft and genetically engineered mouse models of PDAC [68]. However, clinical trials using 7rh have not been undertaken. Additionally, as a central pathway in collagen signaling, inhibition of the FAK pathway may be a way to interrupt the aggressive effects of collagens on PDAC biology. Currently, two dose-finding phase I trials are investigating the small molecule FAK inhibitor Defactinib in advanced PDAC (NCT02546531, NCT02758587). 

## 8. Future Perspectives

Modulating the PDAC stroma bears the potential to not only ameliorate PDAC cell biology itself but also to increase the amenability of PDAC cells to conventional cytotoxic and radiotherapy. Breaking the chemo- and radiotherapy barrier could therefore possibly result in a significant increase of therapeutic options for patients with PDAC. However, since PDAC is characterized by a multitude of different mutations, pathway alterations, and genetic heterogeneity [153], both future clinical trials and therapies will have to be coupled with the thorough molecular characterization of patients to ensure optimal therapeutic efficacy.

## 9. Conclusions

The ECM possesses fundamental tumorigenic features and is a major factor in both promoting PDAC progression and restricting the delivery of antitumor therapy. Whether by regulating migration, proliferation, antiapoptosis, or cell metabolism, the ECM has a major hand in shaping the hallmarks of cancer in PDAC. Through various biochemical and biomechanical signaling pathways, the ECM creates a niche that directs the fate of PDAC cells. However, the ECM is a complex network of molecules having both pro- and antitumorigenic effects, and depletion of the ECM can have disastrous effects on survival. Therefore, remodeling the balance of these factors as opposed to eradicating the ECM could be a viable strategy for improving outcomes in PDAC. While the initial results of ECM-targeted drugs appear promising, intensified research is required to characterize further therapeutic targets in the ECM.

## Figures and Tables

**Table 1:** Effects of collagens on Pancreatic ductal adenocarcinoma (PDAC) cells.

Type of Collagen	Effect on PDAC Cells	Promotion (+)/Inhibition (−)
**Collagen I**	Apoptosis	−
	EMT	+
	FAK pathway	+
	Histone acetyltransferases	+
	Migration	+
	MMP	+
	Proliferation	+
**Collagen IV**	Migration	+
	Proliferation	+
**Collagen V**	Adhesion	+
	Migration	+
	Proliferation	+
	Viability	+
**Collagen XV**	Migration	−

**Table 2:** Overview of currently active clinical trials on ECM-targeted therapy.

**HA Degrading Enzymes**			
**PEGPH20**	**Phase**	**Stage**	**Design**
NCT02910882	II	LAPC	PEGPH20 + GEM + Radiation
NCT01959139	I/II	Metastatic	PEGPH20 + FOLFIRINOX vs. FOLFIRINOX
NCT03193190	I/II	Metastatic	GEM/nab/mFOLFOX6 vs. Atezolizumab + Cobimetinib vs. Atezolizumab + PEGPH20 vs. Atezolizumab + BL-8040
NCT03481920	I	LAPC/Metastatic	PEGPH20 + Avelumab (single arm)
NCT01839487	II	Metastatic	PEGPH20+ GEM/nab vs GEM/nab
**Angiotensin inhibitors**			
**Losartan**	**Phase**	**Stage**	**Design**
NCT01821729	II	LAPC	Losartan + FOLFIRINOX + Proton Beam Radiation (single arm)
**Vitamin D receptor agonists**			
**Paricalcitol**	**Phase**	**Stage**	**Design**
NCT03520790	I/II	Metastatic	GEM/nab + Placebo vs. GEM/nab + Paricalcitol
NCT03415854	II	Metastatic	Paricalcitol + Cisplatin + GEM/nab (single arm)
NCT02930902	I	Resectable	Pembrolizumab + Paricalcitol vs. Pembrolizumab + Paricalcitol+ GEM/nab
NCT03331562	II	Metastatic	Pembrolizumab + Paricalcitol vs. Pembrolizumab +Placebo
NCT03300921	I	Resectable	Pembrolizumab + Paricalcitol vs. Pembrolizumab + Placebo
NCT03519308	I	Resectable	Nivolumab + GEM/nab + Paricalcitol vs. Nivolumab + GEM/nab
**Retinoic acid receptor agonists**			
**ATRA**	**Phase**	**Stage**	**Design**
NCT03307148	I	LAPC/Metastatic	ATRA + GEM/nab (single arm)
**Macropinocytosis inhibitors**			
**Hydroxychloroquine**	**Phase**	**Stage**	**Design**
NCT01978184	II	Resectable	Hydroxychloroquine + GEM/nab vs. GEM/nab
NCT03344172	II	Resectable	GEM/nab + Hydroxychloroquine + Avelumab vs. GEM/nab + Hydroxychloroquine
NCT01506973	I/II	Metastatic	Hydroxychloroquine + GEM (single arm)
NCT01494155	II	Resectable	Hydroxychloroquine + Capecitabine + Radiation (single arm)
NCT01128296	I/II	Resectable	Hydroxychloroquine + GEM (single arm)
**FAK inhibitors**			
**Defactinib**	**Phase**	**Stage**	**Design**
NCT02758587	I/II	LAPC/Metastatic	Defactinib + Pembrolizumab (single arm)
NCT02546531	I	LAPC/Metastatic	Defactinib + Pembrolizumab + GEM (single arm)

LAPC, locally advanced pancreatic cancer; GEM, Gemcitabine; GEM/nab, Gemcitabine + nab-Paclitaxel; HQ, Hydroxychloroquine.
